# Identification of several high-risk HPV inhibitors and drug targets with a novel high-throughput screening assay

**DOI:** 10.1371/journal.ppat.1006168

**Published:** 2017-02-09

**Authors:** Mart Toots, Mart Ustav, Andres Männik, Karl Mumm, Kaido Tämm, Tarmo Tamm, Ene Ustav, Mart Ustav

**Affiliations:** 1 Institute of Technology, University of Tartu, Tartu, Estonia; 2 Icosagen Cell Factory OÜ, Ülenurme vald, Tartumaa, Estonia; 3 Institute of Chemistry, University of Tartu, Tartu, Estonia; 4 Estonian Academy of Sciences, Tallinn, Estonia; University of Wisconsin Madison School of Medicine and Public Health, UNITED STATES

## Abstract

Human papillomaviruses (HPVs) are oncogenic viruses that cause numerous different cancers as well as benign lesions in the epithelia. To date, there is no effective cure for an ongoing HPV infection. Here, we describe the generation process of a platform for the development of anti-HPV drugs. This system consists of engineered full-length HPV genomes that express reporter genes for evaluation of the viral copy number in all three HPV replication stages. We demonstrate the usefulness of this system by conducting high-throughput screens to identify novel high-risk HPV-specific inhibitors. At least five of the inhibitors block the function of Tdp1 and PARP1, which have been identified as essential cellular proteins for HPV replication and promising candidates for the development of antivirals against HPV and possibly against HPV-related cancers.

## Author summary

Human papillomaviruses are causative agents of many different cancers; they are most commonly associated with cervical cancer which leads to about quarter of a million deaths each year. Regardless of extensive studies for decades there is no specific cure against HPV infection. During this research, we have engineered modified HPV marker genomes that express Renilla luciferase reporter gene which expression level correlates directly with viral genome copy number. We have used such modified HPV genome in high-throughput screening of NCI Diversity Set IV chemical library and have identified a number of novel high-risk HPV-specific chemical compounds and drug targets. Such Renilla-expressing marker genomes could be used in various cell systems suitable for HPV replication studies to conduct high-throughput screens and quantify viral genome copy number quickly and effectively.

## Introduction

Human papillomaviruses (HPVs) are small, double-stranded DNA viruses that infect the epithelium of the skin and mucosa. To date, at least 202 HPVs have been characterized, but studies suggest that the true number is considerably higher[1,2]. HPVs induce benign lesions in the mucosal and cutaneous epithelia, and most of the infections are cleared by the immune system within a year after infection. However, a small fraction of infections become persistent and may lead to the transformation of cells and the development of invasive cancers. The vast majority of HPV-associated cancer cases are related to oncogenic mucosal high-risk HPVs from genus alpha (types 16, 18, 31, 33, 35, 39, 45, 51, 52, 56, 58, 59, and 68); types 16, 18, 31, 33 and 45 are estimated to cause more than 99% of cervical cancers [3–5]. Cervical cancer was the seventh most common cancer and the fourth most common among women in the year 2012. There are an estimated 528,000 new cases per year, with 80% in developing countries, leading to 266,000 deaths[6]. In the United States alone, 6 million new HPV cases are diagnosed every year[7]. In addition to alpha PVs, infection with cutaneous beta PVs is also prevalent in the population. These viruses have not been as thoroughly studied as alpha PVs, but an increasing number of studies suggest their association with skin cancer[8,9]. In addition to serious health problems, HPV-related infections and cancers are a serious economic burden: in the United States, a total of 3.4 billion is spent annually on the diagnosis and treatment of HPV-related cancers, which does not even account for the cost for treating various warts and other benign papillomas[10]. These numbers suggest that there is a clear need for better prevention and treatment solutions regarding HPV-related diseases.

Regardless of being studied for decades, there is still no effective cure for an ongoing HPV infection. There are approved invasive treatments, such as cryotherapy, larger excision procedures, laser therapy and electrosurgery, which do not eliminate HPV DNA completely, leading to a 40% chance of recurrence of infection[11,12]. Immune system stimulants (imiquimod for example) as well as trichloroacetic acid and podophyllotoxin have 50% efficiency and a relatively high recurrence rate[13,14]. In addition to therapy, three vaccines against HPV are available: Gardasil (against subtypes 6, 11, 16 and 18), Gardasil 9 (against subtypes 16, 18, 31, 33, 45, 52, 58, 6 and 11) and Cervarix (against subtypes 16 and 18). These vaccines have proven to be very useful tools in the prevention of HPV infections[15,16], but they are prophylactic, and their availability is limited, especially in developing regions, which have the highest cervical cancer prevalence[17].

Although no effective HPV inhibitors have been developed, several compounds and targets have been analyzed. E1 and E2 are the only two viral proteins necessary for HPV genome replication. The first attempts in the development of HPV inhibitors were focused on E1, specifically targeting its ATPase and DNA helicase activities[18,19]. These inhibitors were never approved, presumably due to a lack of specificity for E1. For efficient replication, E1 interacts with E2, which directs it to the origin of replication. As the crystal structure of the E1-E2 complex has been described[20], several compounds—the first HPV-specific compounds, inhibiting complex formation, have been developed[21,22]. While these inhibitors effectively reduced HPV replication, they were only effective against low risk HPV types 6b and 11. It is known that both E1 and E2 interact with numerous cellular (replication) proteins, and these interactions are crucial for successful HPV genome replication, gene expression regulation and maintenance. Therefore, designing molecules specifically targeting these interactions would perhaps lead to the desired goal (reviewed extensively in[23]). Or perhaps HPV even uses pathways based solely on cellular proteins to facilitate its infection, which are essential for the virus but dispensable for the cells (see one example in the Results section below).

In addition to targeting HPV proteins, the viral genome itself could be the target as well. Several sequence-specific DNA binding compounds belonging to the pyrrole-imidazole polyamide class have been developed[24,25]. This type of compounds bind to AT-rich regions near the E1 and E2 binding sites in the HPV replication origin and effectively reduce the stably maintained episomal viral genomes, presumably by affecting the binding of the E1-E2 complex to the origin.

The current situation in HPV therapy strongly suggests that there is a need for specific drugs targeting HPV infection, preferably without restriction to specific subtypes.

HPV infects epithelial cells in the cutaneous and mucosal epithelia, and its life cycle is dependent on keratinocyte differentiation. Thus, most of the work regarding the HPV life cycle has been conducted in human primary epithelial keratinocytes; using these cells is relatively time-consuming and expensive[26–31]. This is particularly true for drug development, since currently, high-throughput screening (HTS) of available chemical libraries is a widely used technique to identify new inhibitors of various diseases[32]. There are number of systems for conducting HTS to target the HPV life cycle. Fradet-Turcotte et al. have developed a model system where they use heterologous E1 and E2 expression vectors and monitor the replication of an HPV origin-containing plasmid using luciferase as a reporter[33]. This model system is definitely useful in the identification of novel drugs. However, it only allows researchers to study the E1- and E2-dependent initiation of replication, and since it does not use the full HPV genome as model, it does not take into account the functions of other HPV proteins as well as the regulation of viral gene expression, maintenance, vegetative amplification and segregation. Moreover, as E1 and E2 are expressed from heterologous vectors, both protein and replication levels may significantly differ from HPV genome replication. Two other model systems based on HaCat cells have also been developed. One of them allows researchers to monitor the stable maintenance of the *wt* HPV11 genome, but it can only be used for a limited number of passages[34]. Another HaCat cell-line system uses HPV VLPs that express different reporter genes. The cells are infected with these VLPs, and reporter gene expression can be monitored. This system is extremely powerful when screening compounds inhibiting the very early stages of HPV infection[35].

U2OS cells, which are derived from moderately differentiated osteosarcoma, have an adherent epithelial morphology and carry wild-type p53 and pRB genes, which have proven to be useful for studying various aspects of the HPV life cycle[36–38]. It has been shown that the replication mechanism of HPVs in U2OS cells is identical to other cell lines, such as HaCat cells[39,40]. In addition to describing replication, transcriptome analyses of mucosal HPVs 11 and 18 and cutaneous HPV 5 have been published[41–43]. These studies indicate that HPV gene expression in the U2OS cell line is very similar to that in keratinocytes[44,45]. Replication and transcriptome studies suggest that U2OS cells provide an adequate cellular environment to study HPV, and since they can be rapidly and cost-efficiently grown, they would be useful in conducting high-throughput screens to identify novel HPV inhibitors.

In this study, we report the generation of various HPV 18, 16 and 5 marker genomes containing reporter genes for rapid and easy quantification of viral copy number. First- and second-generation marker genomes contained reporter genes in various configurations in the late region of the HPV genome. All of these versions had reduced replication ability. Based on the transcription map of HPV18[41], we next turned to the early region of the HPV genome and added the Renilla luciferase gene into the ORF of E2 immediately after the overlapping region of E1 and E2, which added 20 (in the case of HPV5 and 16) or 22 amino acids (in the case of HPV18) at the N-terminus of the luciferase protein. In the C-terminus, luciferase carried the 2A peptide sequence of FMDV and was followed by the complete sequence of the full-length E2 protein starting from the methionine. This configuration resulted in the production of two functional proteins during the translation of the fusion mRNA: full-length E2 and functional Renilla luciferase with the short E2 protein 20- or 22-amino-acid tag at the N-terminus. Since the expression levels of Renilla luciferase are controlled by viral transcription, its expression level correlates with the HPV copy number. In addition to validating this novel model system, we used it for HTS of the NCI Diversity Set IV public chemical library and a customized library and identified several novel high-risk HPV-specific inhibitors with IC50 values ranging from 2.5–60 μM. These inhibitors effectively blocked the replication of HPV18, HPV16, HPV31, HPV33 and HPV45 but not HPV11 or HPV5.

## Materials and methods

### Cell lines and transfection

U2OS cells, which were obtained from the American Type Culture Collection (ATCC no: HTB-96), the modified cell lines U2OS GFP2-Fluc #10.15, U2OS-EBNA1 (Icosagen Cell Factory Ltd) and the HPV18 Rluc-E2-positive U2OS #10.15 subclones #2G10 and #2B3, were grown in Iscove’s modified Dulbecco’s medium (IMDM) supplemented with 10% fetal calf serum (FCS). The U2OS cells were transfected with the indicated amounts of different HPV minicircles or 500 pmol of HPV18-specific siRNAs by electroporation (220 V, 975 μF) with a Bio-Rad Gene Pulser II that was supplied with a capacitance extender (Bio-Rad Laboratories). The U2OS GFP2-Fluc #10.15 cell line was generated by transfecting U2OS cells with a linearized expression vector containing both the GFP2 and Firefly luciferase (Fluc) ORFs as well as a puromycin resistance gene. Individual puromycin-resistant clones were picked and analyzed by GFP and Fluc expression. HPV18 Rluc-E2 positive subclones were generated similarly to those described in[36]. The CIN 612E cells (kind gift from Dr. Frank Stubenrauch) were grown in Keratinocyte-SFM Medium Kit (Gibco, cat# 17005075).

### Plasmids

The parental plasmid pMC-HPV18 was constructed for the production of HPV18 genome miniplasmids. A recognition site for the BglII restriction endonuclease was introduced into the HPV18 genome between nt 7473 and nt 7474 (herein, the numbering of the HPV18 genome is according to the NCBI Reference Sequence NC_001357.1). These sites were used previously without observing changes in gene expression compared to unaltered HPV18[44]. The modified HPV18 genome was cloned into the minicircle production plasmid pMC.BESBX. The pMC backbone, derived from pMC.BESBX, permits the production of the HPV18 genome from pMC-HPV18 as a supercoiled minicircle that contains a 92-bp-long non-HPV sequence. pMC-HPV5 was generated by linearizing the HPV5 genome with XmaJI (site in L2) and cloning the DNA into the XbaI site in the pMC.BESBX vector. pMC-HPV16 was made by inserting the BamHI-linearized HPV16 genome (site in L1) into the BglII site in the pMC.BESBX vector. pMC-HPV33 was cloned into the pMC.BESBX vector SalI sites introduced into the HPV33 genome after nt. 6915. pMC-HPV45 was cloned into the pMC.BESBX vector using the BglI sites introduced into the HPV45 genome after nt. 7569. Miniplasmid production was performed in E. coli strain ZYCY10P3S2T according to a previously published protocol[46]. Finally, the HPV genomes were purified from E. coli as supercoiled minicircles using the QIAfilter Plasmid kit (Qiagen). A vector containing the HPV31 genome sequence was obtained from the International Human Papillomavirus Reference Center, digested with EcoRI and religated before the transfection as described in[47]. The first-generation HPV18 marker genomes were constructed by substituting the late region of the genome different expression marker genes (Renilla luciferase, Rluc; Gaussia luciferase, Gluc; red fluorescent protein, RFP; and destabilized RFP, TurboRFP-dest1) driven by the Rous Sarcoma Virus (RSV) LTR promoter. The second-generation HPV18 marker genomes were constructed by introducing the marker gene Renilla luciferase into the L2 ORF. A full-length VCIP IRES element that has been demonstrated to be active in U2OS cells[48] was added in front of the Rluc marker gene to promote its translation, as described in[49]. Two variants of the second-generation marker genome were constructed that differed in the absence or presence of the heterologous polyadenylation region after the Rluc coding sequence (HPV18L2-Rluc, HPV18L2-Rluc-pA). The HPV18 Rluc-E2 marker genome was constructed by adding the Renilla luciferase-encoding cDNA (from pRL-TK, Promega) with the C-terminal 2A sequence of FMDV between the E1 and E2 ORFs. The length of the overlapping region between the 3’ end of the E1 ORF and the 5’ end of the E2 ORFs in HPV18 is 71 nt (nt 2818–2887). This resulted in a novel ORF of a single polypeptide that starts from the native start codon of E2. The HPV5 marker genome HPV5-RlucE2 and the HPV16 marker genome HPV16-RlucE2 were generated similarly to HPV18-RlucE2. The HPV18-RlucE2 K490A marker genome contains a mutation (amino acid lysine 490 is mutated to alanine in the E1 protein) that abrogates the E1 protein ability to hydrolyze ATP. This results in replication-deficient viral genome [50]. This mutant genome was generated by PCR mutagenesis using primer CTTAGTATCTGTTAACGGTTCCAACCAAAAATGACTAGTGGAATTCACAAATGATATTACTGCTCCTTGTATAAAGTGTATAAAACTCATTCCAAAATATGAcgcTCCTGTATTTGC. The expression vectors for HPV18 E1 and E2 and the origin-containing HPV18-URR minicircle plasmid are described in[39]. The Epstein Barr Virus oriP plasmid p994 was a kind gift from B. Sugden, described in[51]. HPV18 E5-, E6-, E7- or E6-E7- mutant genomes and their replication properties were described in [39]. ShRNA expression was under the control of RNA polymerase III promoter U6.

Tdp1 shRNA sequence: GCACGATCTCTCTGAAACAAACTCGAGTTTGTTTCAGAGAGATCGT

PARP1 shRNA sequence: GGACTCGCTCCGGATGGCCTTCAAGAGAGGCCATCCGGAGCGAGTCC

Tdp2-1 shRNA sequence: GTACAGCCCAGATGTGATACGAATATCACATCTGGGCTGTAC

Tdp2-2 shRNA sequence: GAAGGATATTTCACAGCTACGAATAGCTGTGAAATATCCTTC

### High-throughput screen for identification of HPV18 inhibitors

The U2OS-GFP-Fluc #10.15 cells were transfected with 2 μg of the HPV18-Rluc-E2 minicircle, and the cells were seeded onto 100 mm plates. On the next day, the cells were detached and seeded onto 96-well plates (5000 cells per well). Forty-eight hours after the transfection, the screened compounds were added to the media in 5 μM and 1 μM concentrations. The cells were grown for three days, and both Firefly luciferase (shows cellular viability) and Renilla luciferase (shows HPV copy number) were measured using the Dual-Glo luciferase assay system (Promega) according to manufacturer’s protocol with the GloMAX-96 luminometer (Promega). The results were blotted on a XY-scatter diagram, and HPV-specific hits were chosen.

### Chemicals

The Diversity set IV and the additional compounds NSC9782, NSC 88915, NSC 82269, NSC 109128 and NSC 305831 were obtained from the Drug Synthesis and Chemistry Branch, Developmental Therapeutics Program, Division of Cancer Treatment and Diagnosis, National Cancer Institute. Camptothecin (CPT) (sc-200871) and ABT-888 (sc-202901) were purchased from Santa Cruz Biotechnology.

### HPV copy number quantitation

The viral genome copy number in U2OS cells during replication was analyzed by quantitative real-time PCR (qPCR). Genomic DNA was extracted from U2OS cells, and the samples were linearized by digestion with the appropriate enzyme (see above), and the bacterially produced input DNA was fragmented by digestion with DpnI. For each qPCR reaction, 3 ng of DNA was used, and the reactions were performed with EvaGreen qPCR Mix Rox (Solis BioDyne) according to the manufacturer’s protocol on a 7900 HT Fast Real-Time PCR System (Applied Biosystems). The HPV18 replication signal was amplified with the following oligonucleotides (300 nM of each per reaction): 5’-GCGCTTTGAGGATCCAAC-3’ and 5’-GTTCCGTGCACAGATCAG-3’. For HPV5, the following oligonucleotides were used: 5’-GGTTGCAGGAACTGTGAGGT-3’ and 5’-TCCGCGACAGTCGGGGCACAGG-3’. For HPV16, the oligonucleotides were 5’- CCCACAGCTACAGATACAC-3’ and 5’- GCAGGTGTGGTATCAGTTG-3’. The analysis was performed according to the comparative threshold cycle (ΔCt) method. The results were calculated from the PCR cycle number in which the HPV signal exceeded the threshold value (Ct_HPV_). The Ct_rDNA_ was detected as a normalization standard from the ribosomal DNA gene sequence in the U2OS genome with the following oligonucleotides (300 nM of each): 5’- GCGGCGTTATTCCCATGACC-3’ and 5’- GGTGCCCTTCCGTCAATTCC-3’. The relative value C_N_, which reflects the average viral genome copy number per cell, was calculated from the data with the formulas ΔCt = Ct_HPV_ − Ct_rDNA_ and C_N_ = 2^-ΔCt^.

### Transient replication assay

For first- and second-generation marker genome analyses, episomal DNA was extracted as described in[52]. For Southern blot analyses, U2OS, U2OS#10.15 or U2OS-EBNA1 cells were transfected with 2–5 μg of the indicated HPV genome or 1 μg of oriP plasmid (only in the case of U2OS-EBNA1 cells). HPV DNA was linearized and digested with DpnI to cleave non-replicated, bacterially produced DNA. The samples were resolved in an agarose gel, blotted, and hybridized with an HPV genome or oriP sequence-specific probe labeled with [α-^32^P]dCTP using random priming (DecaLabel kit; Thermo Scientific). Specific HPV replication signals were detected by Typhoon TRIO (GE Healthcare).

### Measurement of Firefly and Renilla luciferases

The cells were lysed with passive lysis buffer by freezing and thawing. Both Renilla and Firefly luciferase expression was measured with a Glomax 20/20 luminometer using the Dual-Luciferase reporter assay system following the manufacturer’s protocol (all equipment and components from Promega Corporation).

### Western blot

The cells were lysed with Laemmli buffer (4% SDS, 20% glycerol, 120 mM Tris-Cl (pH 6.8), and 200 mM DTT) and boiled for 10 minutes at 100°C. The samples were resolved in an SDS-PAGE gel and transferred to Immobilon-P PVDF membrane (Millipore). Tdp1 and PARP1 were detected with their specific antibodies from Santa Cruz Biotechnology: sc-365674 and sc-56197, respectively. Tubulin (used as a loading control) was detected using Sigma Aldrich antibody T9026. Anti-mouse peroxidase-conjugated secondary antibody (LabAS) and Amersham ECL Western blotting Detection Kit (GE Healthcare) were used for visualization. Signals were exposed on an X-ray film (AGFA).

### Cell cycle analyses

The cells were detached and collected by centrifugation 300g 5 minutes. The pellet was resuspended 100μl 1X PBS and 900μl ice-cold 80% ethanol was added drop-wise. The cells were next incubated on ice for at least 30 minutes, collected by centrifugation, resuspended in 1X PBS containing 50μg/ml propidium iodide and 200μg/ml RNase A and incubated at 37°C for 45 minutes. Cell cycle profiles were analyzed by flow cytometry (LSR II from Becton Dickinson) and by FlowJO 10 software using Watson (pragmatic) model.

## Results

### Generation of U2OS cell line and HPV marker genomes suitable for high-throughput screening of chemical libraries

**Fig 1:**
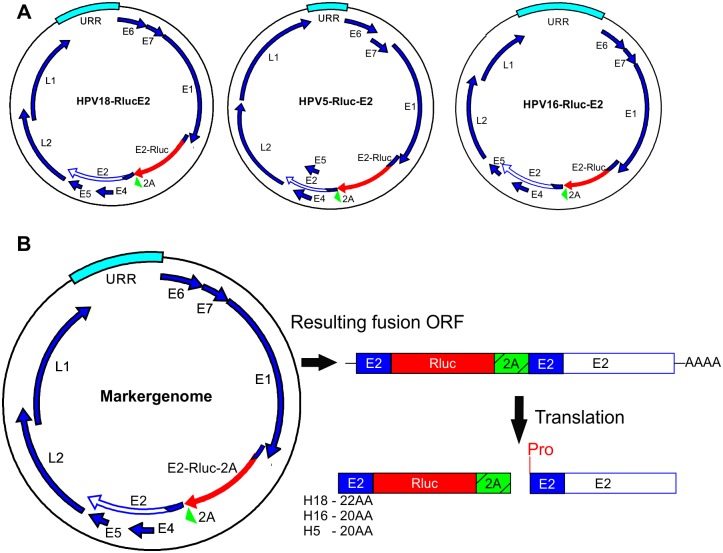
Schematic map and the working principle of the Renilla luciferase-encoding marker genomes. The coding sequences of Renilla luciferase and the 2A peptide of FMDV are inserted in frame into the E2 ORF of HPV immediately after the overlapping region of E1 and E2, and the full-length E2 gene follows. Translation results in functional Renilla luciferase and a full-length E2 proteins separated by the “cleavage” of the 2A peptide. The Renilla luciferase protein contains additional amino acids of the N-terminal E2 protein, as shown. The 20–22 amino acids in the N-terminus of E2 protein are thus present in both the Renilla protein as well as the full-length E2 protein. Due to the effects of 2A peptide, an additional amino acid proline is present in the beginning of the E2 protein. Renilla luciferase expression is controlled by viral transcription. Modified regions of the viral genome are shown in red and green.

**Fig 2:**
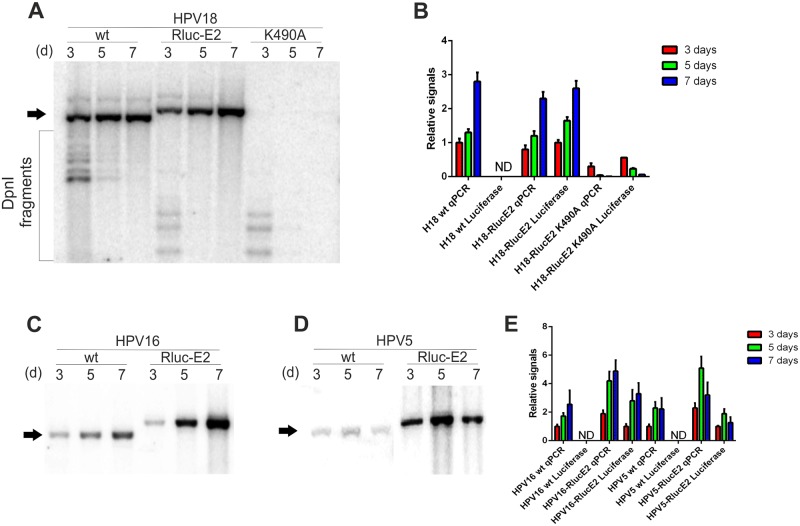
Analysis of the transient replication of HPV18, HPV16 and HPV5 marker genomes in the U2OS GFP2-Fluc #10.15 cell line. U2OS #10.15 cells were transfected with 2 μg of HPV18 wt, HPV18-RlucE2, HPV18-RlucE2-K490A, HPV16 wt, HPV16-RlucE2, HPV5 wt and HPV5-RlucE2. The genomic DNA was extracted 3, 5 and 7 days after transfection, the HPV DNA was linearized and bacterially produced input DNA was digested with DpnI. Replication signals were visualized by Southern blot, and the position of the replicated DNA is shown with an arrow. Replication signals were also quantitated by qPCR and are expressed relative to either HPV18, HPV16 or HPV5 wt at the 3-day timepoint. Both Renilla (from HPV marker genomes) and Firefly (from U2OS genome) luciferase were measured in a dual-luciferase assay from the same experiments, and the results are expressed as the Rluc/Fluc ratio (Luciferase) relative to the 3-day timepoint from either HPV18-RlucE2 (**A and B**), HPV16-RlucE2 or HPV5-RlucE2 (**C–E)**. *Wt* (and HPV18-RlucE2-K490A) genomes serve as negative controls for Renilla expression. Error bars represent standard deviations from at least two independent experiments.

To evaluate cellular viability and the potential toxic effects of the compounds during the HT screen, we generated a monoclonal U2OS cell line stably expressing both the GFP and Firefly luciferase (Fluc) reporter genes, called U2OS-GFP2-Fluc #10.15. Insertion of these reporter genes allows to measure cell growth and viability as well as HPV genome replication (S1 Fig). To measure HPV genome replication rapidly and quantifiably, we added reporter genes (like luciferase or fluorescent protein) to the viral genome so that their expression would be controlled by viral gene expression. First, we generated first- and second-generation marker genomes (schematic maps in S2 Fig), where modifications were made in the late region of HPV. However, all modified genomes had a reduced replication capacity (S2C Fig). We next turned to the early region of HPV genome and tried to add the Renilla luciferase coding sequence into the ORF of E2. The portion of the ORF of E2 protein encoding the first 22 amino acids of E2 protein overlaps with the ORF of E1 protein in case of HPV18. Thus, the Renilla luciferase reporter gene was added in frame after 22 N-terminal amino acids of the HPV18 E2 ORF, immediately after the overlapping region with E1 ORF. The Renilla luciferase reporter coding sequence was followed in frame by the FMDV 2A peptide sequence, which induces single peptide bond pausing during the translation of the mRNA. The 2A peptide sequence was followed by the full-length E2 protein sequence starting from the E2 methionine. This configuration results in two proteins: the Renilla luciferase containing first 22 amino acids of HPV E2 protein in its N-terminus and the full-length HPV E2 protein. The first 22 amino acids of E2 protein are thus synthesized twice. In the case of HPV5 and HPV16, similar additions were made in frame after 20 N-terminal amino acids of the respective HPV genomes. Schematic representations of the Rluc-E2 marker genomes and their working principle are shown in Fig 1. Detailed descriptions of the marker genomes are shown in the materials and methods section. To characterize the replication properties of the marker genomes, U2OS #10.15 cells were firstly transfected with HPV18 *wt*, HPV18-RlucE2 and HPV18-RlucE2-K490A genomes. Genomic DNA was extracted 3, 5 and 7 days after the transfection, transient replication was analyzed by Southern blot (Fig 2A) and the HPV copy number was quantitated (Fig 2B). It is clear that addition of the Renilla luciferase coding sequence into the ORF of E2 protein did not alter the replication properties of HPV18 significantly compared to the *wt* genome. In the qPCR analyses, ~150bp fragment of HPV genome that contains only one DpnI restriction site, is amplified. The relatively small signal obtained for HPV18-RlucE2-K490A in the qPCR analyses comes likely from the fact that DpnI restriction is not 100%. Both Firefly (from U2OS genome) and Renilla (from HPV marker genomes) luciferase expression were also measured in the same experiment and are presented as the ratio of Rluc/Fluc, as measured by the dual-luciferase assay described in the materials and methods section. The results in Fig 2B show that for the HPV18, Renilla luciferase expression could be used to describe changes in the viral copy number during replication. The *wt* and K490A genomes served as negative controls for Renilla luciferase expression. The replication-deficient K490A marker genome shows relatively high Rluc/Fluc ratio the in 3-day timepoint likely because there is still large portion of transfected, transcriptionally active DNA present in the cells.

The marker genomes of HPV16 and HPV5 were analyzed exactly as HPV18 (Fig 2). In case of HPV16, the marker genome replicated similarly to the *wt* genome, for HPV5, the marker genome replication is 2–3 times more effective than the *wt* genome. Renilla luciferase expression was reproducibly detectable, the net luminescence from 1 second integration time ranged from 10 to 25 million from approximately 400 000 cells. Using HPV18-specific siRNAs, we showed that the expression level of Renilla luciferase correlated with the HPV18 copy number during initial amplification (S3 Fig). We have characterized the transcription map for HPV18 in U2OS cells[41], and the results shown in S4 indicate very similar RACE PCR patterns for *wt* HPV18 and HPV18-RlucE2. This confirms that the promoter activity and splicing in the viral early region of the marker genome are similar to *wt* HPV18.

### Generation and analysis of monoclonal U2OS #10.15 cell lines containing the stable episomal HPV18-Rluc-E2 genome

**Fig 3:**
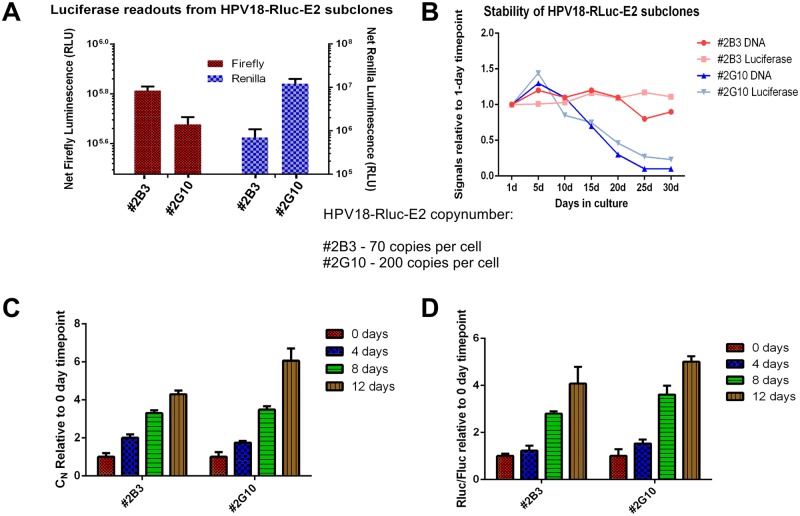
Analyses of stable replication and late amplification of monoclonal U2OS #10.15 cell lines containing an episomal HPV18-Rluc-E2 marker genome. **A:** The expression of Renilla (right axis) and Firefly (left axis) luciferases from two clones of U2OS #10.15 cells stably maintaining an episomal HPV18-Rluc-E2 genome (#2B3 and #2G10), measured on two different days. Renilla expression resembles differences in the HPV18-RlucE2 copy number in these cell lines. Firefly expression resembles the growth of the cells. Viral copy numbers are indicated. **B:** To evaluate the stability of these subclones, #2B3 and #2G10 were thawed from the cell banks generated and cultivated in subconfluent conditions for 30 days. HPV-RlucE2 copy number (by qPCR) and Rluc/Fluc ratios were measured in every 5 days and the values are expressed relative to the 1-day timepoint. **C and D:** To measure late amplification, the cells were seeded in 6-well plates and grown for the indicated number of days without splitting. **C:** To measure viral replication, the genomic DNA was extracted 0, 4, 8 and 12 days after seeding the cells, linearized and quantitated by qPCR. Replication signals obtained from the subclones are expressed relative to the signals obtained from the respective 0-day timepoint. **D:** Both Renilla (from HPV18 marker genome) and Firefly (from U2OS genome) levels from the same experiment were measured in a dual-luciferase assay, and the values obtained from either subclone are expressed relative to the signals obtained from the respective 0-day timepoint.

After initial amplification, the stable maintenance phase of HPV replication is turned on. To study this phase in U2OS cells, stable cell lines carrying episomal HPV genomes must be generated. We generated two monoclonal cell lines (#2B3 and 2G10) based on U2OS-GFP2-Fluc #10.15 that contain an episomal HPV18-RlucE2 genome, as described in[36]. First, both Renilla and Firefly luciferase readouts were measured from these cell lines on two different days. The results in Fig 3A show that both #2G10 and #2B3 indeed express Renilla luciferase, indicating that these cell lines contain the HPV18-Rluc-E2 marker genome and express Firefly luciferase, which could be used to evaluate cell growth and viability. Next, genomic DNA was extracted from these cell lines, and the viral copy number was measured by qPCR analyses. Line #2G10 had approximately 200 copies of HPV18-Rluc-E2 per cell, whereas #2B3 had 70. To further characterize the cell lines, we evaluated their stability to maintain the HPV18-RlucE2 genome. The cells were thawed from the generated cell banks and grown in subconfluent conditions for 30 days; Rluc/Fluc ratios and HPV copy number was measured in every 5 days. As shown in Fig 3B, the #2B3 is stable for at least 30 days while HPV18-RlucE2 genome copy number rapidly decreased from the #2G10 after 15 days of cultivation. It is possible that the high copy-number (200 copies) per cell is the reason of instability. During extensive passaging of the cells, the cells divide relatively quickly. In some HPV-positive U2OS cell clones, the segregation of the viral genome could be less effective resulting in decrease of the HPV copy number during extensive passaging of the cells. Still, both cell lines are suitable for identifying the compounds capable of inhibiting the stable maintenance phase of HPV18.

The third replication stage of HPVs is late amplification, which is triggered by the initiation of keratinocyte differentiation. This stage could be studied using organotypic raft cultures or by inducing keratinocyte differentiation with high concentrations of calcium[53,54]. It has been shown that when U2OS cell lines containing episomal HPV18 are grown in confluent culture for at least three days, late amplification is turned on, and the viral copy number can increase by 10-fold. Although the U2OS cell line is a immortalized cell line, the cultivation in dense cultures likely induces a differentiation-like state as early differentiation marker K10 expression is elevated [36]. The cell lines #2B3 and #2G10 were seeded in 6-well plates (150 000 cells per well) and grown for 4, 8 and 12 days with regular feeding but without passaging of the cells. Both genomic DNA and luciferase samples were collected at each timepoint and used to analyze the viral copy number by qPCR and the expression of the Renilla and Firefly luciferase reporter genes. As seen in Fig 3C, the viral copy number increased by 5-fold, reminiscent of late amplification. Fig 3D shows that the increase in the Renilla expression is very similar to the viral copy number change. In case of late amplification studies, the cells are not passaged and thus the viral genome is not lost from the more unstable #2G10.

Collectively, the data shows that cell lines #2B3 and #2G10 can be used in high-throughput screens to identify compounds capable of inhibiting the stable maintenance and/or late amplification of HPVs.

### Five novel HPV inhibitors identified in high-throughput screening of NCI Diversity set IV

**Fig 4:**
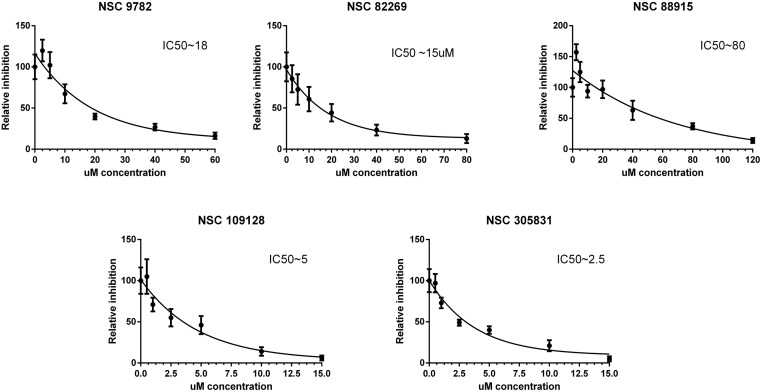
Inhibition of initial amplification of the HPV18 genome by the compounds identified from HTS of NCI Diversity Set IV. U2OS cells were transfected with 2 μg of an HPV18 *wt* minicircle genome and grown in the presence of compounds at the indicated concentrations for 5 days. Genomic DNA was extracted, HPV18 DNA was linearized with BglI and bacterially produced input DNA was digested with DpnI. HPV18 genome replication signals were detected using Southern blot analyses, quantified with a phosphoimager and expressed relative to the vehicle control (DMSO). Logarithmic inhibition curves and approximate IC50 values are shown for each compound. Error bars represent the standard deviation from five independent experiments.

We screened (as described in the Materials and Methods section) NCI Diversity set IV using the model system described in the previous sections for compounds inhibiting the initial amplification of the HPV18 genome in U2OS cells. HT screening was performed with all the compounds in this library in 5 μM and 1 μM concentrations, and 80 compounds were selected for further analyses (approximately 5% of the analyzed compounds). After validation of the hits on the HPV18 *wt* genome replication, five most effective compounds were chosen for detailed analyses (schematic structure and expected activities of the compounds are shown in S7 Fig). To further characterize the compounds, we performed a replication assay, as described in the Materials and Methods section. The inhibition relative to the vehicle control, DMSO, and the logarithmic inhibition curves are shown in Fig 4. All five compounds, NSC 9782, NSC82269, NSC 88915, NSC 109128 and NSC 305831, exhibited concentration-dependent inhibition of HPV18 initial amplification, with IC50 values ranging from 2.5 to 60 μM. To ensure that the inhibition of HPV18 replication is specific, the effects of the compounds on cell cycle progression was evaluated by the exact assay set up described in Fig 4. Both non-transfected and HPV18-transfected U2OS cells were used, the results in S1 Table and S8 Fig clearly demonstrate that the compounds identified here do not alter the cell cycle progression significantly. We additionally analyzed the effect of the compounds on HPV18 ori—URR replication supported by the HPV18 E1 and E2 proteins expressed from heterologous expression vectors. None of the compounds inhibited the replication of URR plasmid facilitated by E1 and E2 expressed from heterologous expression vectors (S5 Fig). These compounds were also analyzed for their ability to inhibit the stable maintenance and late amplification of HPV18 in the U2OS cell line #1.13, which contains episomal viral genomes, as described in[36,41]. Compounds 109128, 305831 and 82269 clearly inhibited both the stable maintenance and vegetative amplification of HPV18, while 88915 only inhibited the stable maintenance phase, and 9782 did not inhibit any of the later replication stages (S6 Fig).

### Tdp1 and PARP1 are involved in HPV initial amplification

**Fig 5:**
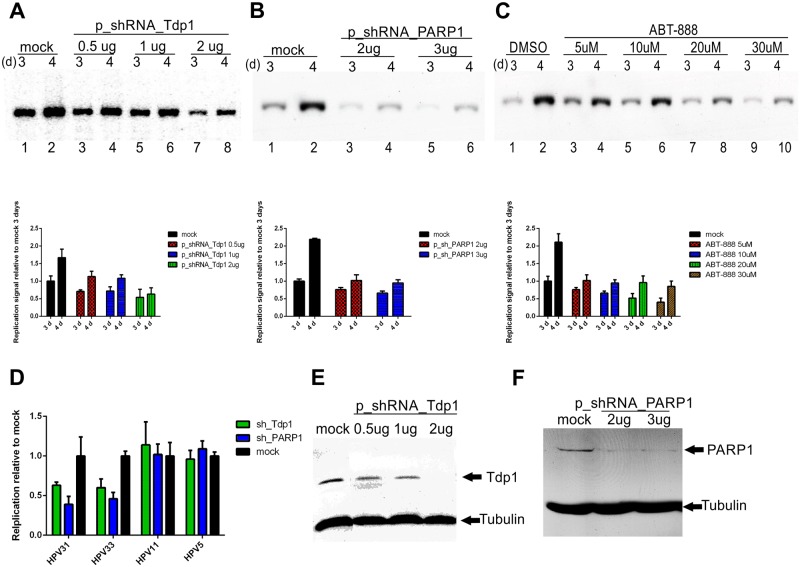
Tdp1 and PARP1 are essential cellular proteins for the initial amplification of the high-risk HPV genome. U2OS cells were transfected with HPV18 *wt* minicircle and an sh_Tdp1 plasmid (**A)** or sh_PARP1 plasmid (**B)**. Genomic DNA was extracted 3 and 4 days after the transfection, linearized and digested with DpnI. The HPV18 replication signal was detected with Southern blot analyses and quantified with a phosphoimager. **C:** U2OS cells were transfected with an HPV18 *wt* minicircle and grown for 3 and 4 days in the presence of different concentrations of the PARP1 inhibitor ABT-888. The HPV replication signal was detected with Southern blot analyses and quantified with a phosphoimager. **D:** U2OS cells were transfected with an HPV31, 33, 11 or 5 *wt* minicircle and the sh_Tdp1 or sh_PARP1 plasmid (2μg). Genomic DNA was extracted 3 days post-transfection, linearized and digested with DpnI The HPV replication signals were detected with Southern blot analyses and quantified with a phosphoimager. **E and F:** Western blot analyses showing the downregulation of the Tdp1 and PARP1 proteins by shRNA at the 3-day timepoint. Empty shRNA vector was used as a mock control for Tdp1 and PARP1 downregulation by sh_RNA plasmids. **G:** Error bars represent standard deviations from two to three independent experiments.

Two of the identified compounds, 88915 and 305831, are known inhibitors of Tdp1[55,56]. To see if Tdp1 is actually necessary for HPV18 initial amplification, we transfected U2OS cells with an HPV18 minicircle genome together with various concentrations of the shRNA_Tdp1 plasmid. Empty shRNA plasmid was used as a mock control. Genomic DNA was extracted 3 and 4 days after the transfection a replication assay was performed and the results quantified. Results in Fig 5A (compare lanes 1 and 2 with 3–8) show at least 50% decrease in HPV18 replication when Tdp1 expression was downregulated.

It was recently shown that the PARP1 protein activates Tdp1 through PARylation and recruits it to sites of DNA damage[57]. PARP1 involvement in initial HPV18 amplification was assessed similarly to Tdp1 using shRNAs or the PARP1-specific inhibitor ABT-888 (Veliparib)[58]. The results in Fig 5 show that similarly to Tdp1, PARP1 is essential for HPV18 replication.

Next, the involvement of Tdp1 and PARP1 in the initial amplification of other HPV types was evaluated by co-transfection of shRNA-s with HPV 31, 33, 11 and 5 minicircle genomes. Genomic DNA was extracted three days post-transfection, analyzed by Southern blot and the signals were quantified (Fig 5D). Unexpectedly, Tdp1 and PARP1 seem to be necessary for high-risk HPV replication only as the downregulation of these proteins decreases the replication of HPV31 and 33 but not 11 and 5.

Effects of Tdp1 and PARP1 downregulation on cell cycle progression were also evaluated in a similar assay setup. Results in S2 Table demonstrate that loss of Tdp1 or PARP1 expression has no significant effect on cell cycle progression of U2OS cells.

It has been previously shown that instead of Tdp1, Tdp2 is actually necessary for HPV replication [25]. In this study, downregulation of Tdp1 actually resulted in increase of the replication. We designed two shRNA-s specific to Tdp2 and firstly analyzed their effect on cell cycle progression of U2OS similarly to Tdp1 and PARP1. The downregulation of Tdp2 significantly altered the cell cycle progression, one shRNA caused the block in G1 and the other one in G2/M phase of the cell cycle (S2 Table). Thus, it was not possible to estimate the potential involvement of Tdp2 in HPV replication in U2OS cells specifically.

### Synergistic effect of Camptothecin (CPT) and HPV18 replication inhibitors

**Fig 6:**
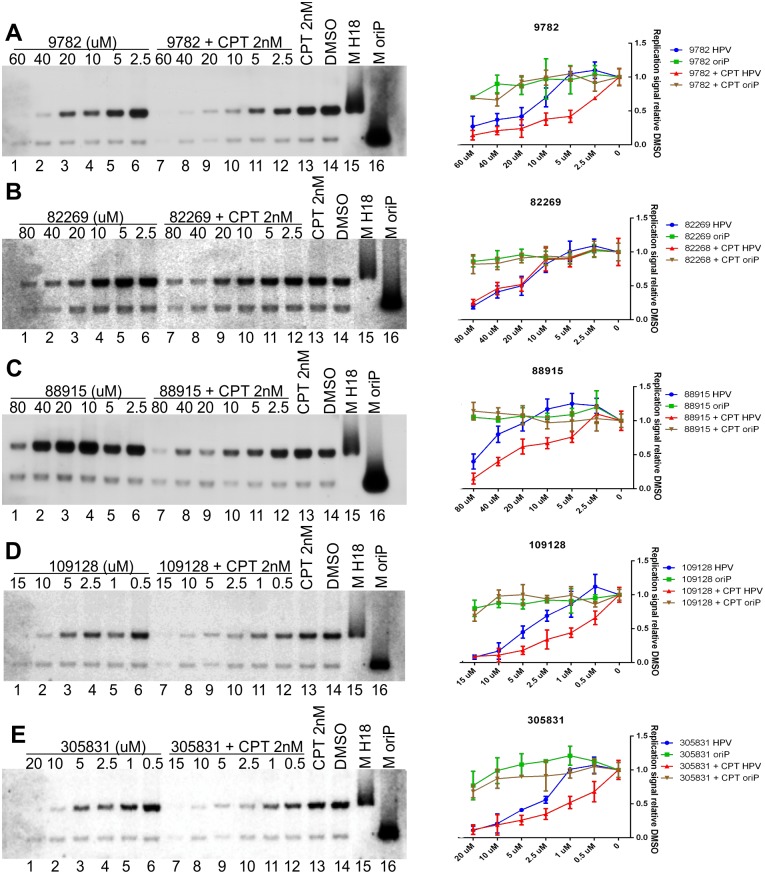
Synergistic inhibitory effect between Camptothecin (CPT) and compounds identified in the HT screen on the initial amplification of the HPV18 genome. U2OS-EBNA1 cells were transfected with HPV18 *wt* and oriP plasmids and grown in the presence of the indicated concentrations of compounds alone or together with 2 nM CPT for 5 days. Genomic DNA was extracted, linearized and digested with DpnI. Both HPV18 and oriP replication signals were detected with Southern blot and quantified using a phosphoimager. Compounds 9782, 88915, 109128 and 305831 showed modest synergistic inhibition together with CPT (panels **A, C, D and E**), whereas compound 82269 did not (panel **B**). Error bars represent standard deviations from at least three independent experiments. Lanes 15 and 16 are size markers for HPV18 and oriP.

CPT is a Topoisomerase I inhibitor that stabilizes entrapped Top1cc complexes on DNA[59,60]. Since Tdp1 is responsible for cleaving entrapped Top1cc complexes from DNA, a synergistic effect occurs between CPT and Tdp1 inhibitors. This type of synergistic treatment between Topoisomerase I inhibitors and NSC 305831 was shown to be effective against Murine Lupus Nephritis significantly improving survival of the infected mice[61]. Topoisomerase I is also shown to interact with HPV E1 and E2 proteins which stimulate its activity to ensure effective replication [62,63] In the experiments shown in Fig 6, we used U2OS cells constitutively expressing the Epstein-Barr virus (EBV) EBNA1 protein—U2OS-EBNA1 cells. The expression of the EBNA1 protein allowed us to monitor the replication of the plasmids containing EBV latent origin—oriP, reminiscent of genomic DNA replication[51,64], [65]. Since HPV amplificational replication is initiated multiple times per cell cycle, it should differ significantly from oriP replication, making the latter suitable as an internal control to determine the specificity of the inhibitors tested. U2OS-EBNA1 cells were co-transfected with an HPV18 minicircle genome, and the oriP plasmid and the cells were grown in the presence of various concentrations of our identified HPV inhibitors alone or together with 2 nM CPT for 5 days, with DMSO used as a vehicle control. A replication assay was performed, and the signals were quantified with a phosphoimager. The results shown in Fig 6 show that all of the HPV inhibitors are specific, as none of them inhibited EBNA1-dependent replication of the oriP plasmid significantly. Treatment with 2 nM CPT alone did not inhibit HPV18 initial amplification. Fig 6 shows that in the case of compounds 9782, 88915, 109128 and 305831, modest concentration-dependent synergistic effects with CPT occurred, as HPV replication was slightly more efficiently inhibited (compare lanes 1–7 with 8–12 in all experiments). Compound 82269 (Fig 6B, compare lanes 1–7 with 8–12), however, showed no synergistic effect together with CPT, suggesting that it inhibits HPV18 replication through some other currently unknown pathway. The compound 9782 acts like a Tdp1 inhibitor but it does not inhibit the stable maintenance and late amplification of HPV18 (S6 Fig) like other compounds do. Perhaps this observation that the compound 9782 is Tdp1 inhibitor is not true, and it inhibits some other protein in this pathway or causes synergistic effects with CPT through some other (unknown) pathway or protein (Tdp2). Tdp2 may be a likely candidate as it has been shown to be capable of carrying out the same functions as Tdp1 [66] The compound 88915 also does not inhibit the late amplification of HPV18 (S6 Fig) although it is a Tdp1 inhibitor. But this could be explained by the low potency of this compound, perhaps very effective inhibition of Tdp1 is necessary during late amplification where HPV genome replication rate is very high.

### Compounds are high-risk HPV inhibitors

Thus far, the experiments for describing the inhibitors were conducted using the high-risk HPV18 genome. Since Tdp1 and PARP1 seem to be necessary for the replication high-risk HPVs but not low-risk or beta HPVs (Fig 5D), we expected the compounds to behave the same. U2OS cells were transfected with HPV types 5, 11, 16, 31, 33 and 45, and the effect of the compounds on these HPV types was evaluated similarly to HPV18. The results in Fig 6 show that indeed the compounds do not inhibit HPV5 or HPV 11 initial amplification. As expected, they are however effective against HR-HPV types 16, 31, 33 and 45.

**Fig 7:**
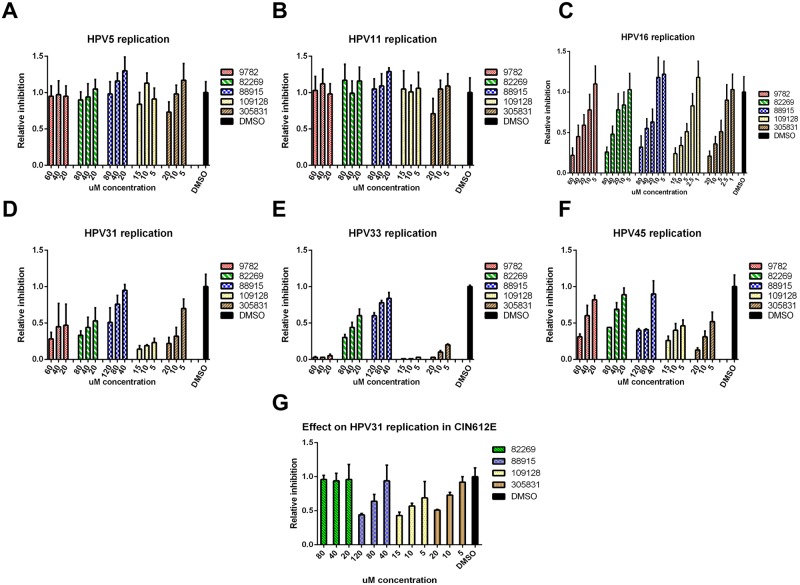
Identified compounds are specific high-risk HPV replication inhibitors. U2OS cells were transfected with various types of HPV minicircle genomes and grown in the presence of the indicated concentrations of the compounds for 5 days. Genomic DNA was extracted, HPV DNA was linearized with the appropriate enzyme for each HPV type and bacterially produced input DNA was digested with DpnI. Replication signals were quantitated by qPCR (for HPV types 5, 11 and 16; panels **A**, **B** and **C**) or from Southern blots using a phosphoimager (for HPV types 31, 33 and 45; panels **D**, **E** and **F**) and are expressed relative to DMSO, as described in the Materials and Methods section. Panel **G:** CIN612E cells were grown in the presence of indicated concentrations of compounds for 6 days and HPV31 replication signal was quantitated from Southern blots using a phosphoimager and are expressed relative to DMSO. Error bars represent standard deviations from two to three independent experiments.

When analyzing chemical compounds, there is always a possibility that the effects may be cell-line specific. In order to ensure that the compounds identified here are true HPV inhibitors, we tested them on HPV31-positive CIN 612E cells (kind gift from Dr. Frank Stubenrauch). The CIN 612E cells were seeded onto 6-well plates and grown in subconfluent conditions in the presence of compounds for 6 days. Genomic DNA was extracted, analyzed by Southern blot and quantified by Phosphoimager. Results in Fig 7G demonstrate that the compounds NSC 88915, NSC 109128 and NSC 305831 decrease the HPV31 copy-number in a concentration-dependent manner in CIN 612E cells. The compound NSC 82269 did not have an effect on HPV31 replication in these cells and the compound NSC 9782 was extremely toxic.

Taken together, these data suggest that the inhibitors identified in this study are specific HR-HPV inhibitors, blocking the replication of HPVs that cause more than 99% of all cervical cancer cases worldwide.

## Discussion

U2OS cells, an osteosarcoma cell line, are suitable for studying the replication properties of various types of HPVs from different phylogenetic genera[36,37]. Studies performed in our lab show that the transcriptomes of both alpha and beta papillomaviruses are very similar to that described in keratinocytes. Therefore, U2OS cells seem to be an adequate model for studying replication, gene expression and other aspects of the HPV life cycle[67,68].

There are numerous well-characterized chemical compound libraries available, meant for the identification of novel drug candidates via high-throughput screening using appropriate model systems. To date, there was no such system for analyzing the complete HPV genome replication cycle. Thus, the first major goal for this work was to construct a model system for HPV research. It has been shown that HPV genomes that lack the late region replicate similarly to *wt* genomes[36]. Therefore, our initial attempts at constructing modified HPV genomes containing easily measurable reporter genes were focused on substituting the late genes of the genome with genes encoding either fluorescent proteins or various luciferases (first- and second-generation marker genomes described in S2A and S2B Fig). The results demonstrated that insertion of the reporter gene cassettes into the late region of the HPV18 genome greatly interferes with gene expression and/or replication properties, as the replication of these marker genomes was almost undetectable (S2C Fig).

Based on the transcriptome analyses of HPV18 in U2OS cells[41], we next tried to insert the Renilla luciferase coding sequence into the beginning of the ORF of the E2 protein and generated marker genomes for HPV18, HPV16 and HPV5 (Fig 1). The coding sequence of Renilla luciferase is inserted into the ORF of the E2 protein directly after the overlapping region of the E1 ORF so that the expression of Renilla luciferase is regulated by the modulation of viral promoters driving the transcription of E2 protein mRNAs. The transcription of Renilla luciferase begins with the native E2 start codon and proceeds for 22 amino acids for HPV18 or for 20 amino acids for HPV16 and 5 before coding the Renilla luciferase. The mRNA produced contains the beginning of E2, the Renilla luciferase and FMDV 2A followed by the full-length E2 (the principle is described in Fig 1). This configuration results in the translation of two functional proteins due to the FMVD 2A protein-induced ribosome skipping: Renilla luciferase that also contains 20–22 N-terminal amino acids of the E2 protein and full-length E2 protein with an additional amino acid proline in its N-terminus. The HPV18 and HPV16 marker genomes replicate slightly more efficiently than the respective *wt* genomes (Fig 2), perhaps due to the more stabilized E2 protein (due to the additional proline in the N-terminus). The HPV5 marker genome replicated at a considerably higher level that the *wt* genome (Fig 2). Since gene expression is different between alpha and beta papillomaviruses, addition of the Renilla coding sequence into the HPV5 E2 ORF may enhance splicing or change the ratios of different viral transcripts, leading to higher expression levels of the E1 and E2 proteins. Nonetheless, to our knowledge, this system is the first suitable system for HTS for beta papillomavirus inhibitors. In addition to analyses of initial amplification, U2OS cells, more specifically cell lines harboring episomal HPV genomes, also allow to study the stable maintenance and late amplification of HPV. For that, stable cell lines containing episomal HPV18 marker genomes were generated (Fig 3). Analyses of these cell lines showed that regardless of the insertion of the Renilla luciferase coding sequence, the HPV18 marker genome displays similar replication characteristics as the *wt* genome during the stable and late amplification phases, at least in U2OS cells. The previously described systems for HPV drug development usually model very specific steps during viral infection (E1-E2 interaction, initiation of viral replication), which significantly reduces the probability of identifying potential inhibitors. As the model system described here allows to study all three HPV genome replication stages and harbors the expression of all viral proteins, more suitable drug targets and inhibitors could be identified, and new mechanistic insights into HPV pathogenesis could be revealed.

Although there are currently three vaccines against various types of HPVs that effectively prevent infection with various HPV types, no approved effective cure for an ongoing infection is available. Mostly identification of novel drugs against various targets begins with high-throughput screening of chemical libraries containing thousands of compounds[69]. Thus, using the model system described here, we conducted a HTS of the NCI Diversity Set IV public chemical library, which consists of different classes of compounds that have shown some type of biological activity. The first round of screening gave ~80 positive hits, out of which 5 compounds (the structures of the compounds is shown in S7 Fig) inhibited HPV18 initial amplification in the low-micromolar range (Fig 4). Many studies regarding HPV replication have been carried out by measuring the E1- and E2-dependent replication of an HPV URR plasmid, which contains the origin of replication. Even an HTS model system for measuring URR replication has been developed[33]. This type of model uses heterologous expression vectors for E1 and E2 expression and could be used for studying the E1/E2-dependent initiation of HPV replication. However, the compounds identified in this study did not inhibit the replication of a URR-containing plasmid (S5 Fig), and compound 88915 even slightly activated the URR plasmid replication. It is possible that this compound stimulates cell growth, prolongs the S-phase or stabilizes replication proteins. Therefore, to identify HPV replication inhibitors, it is important to analyze full-length HPV genome replication because it includes the expression of other viral proteins besides E1 and E2, appears to include different cellular proteins and/or uses different mechanisms for replication.

In almost all HPV-related cancers, the viral genome is integrated into the host genome. Integration of the viral genome usually occurs during persistent infection, and thus to prevent integration and tumor progression, already-established infections must be targeted. Therefore, we next tested these compounds on the stable maintenance and late amplification of HPV18. Four out of five compounds successfully inhibited the stable maintenance phase of viral replication (S6A Fig), and three compounds inhibited late amplification as well (S6B Fig).

**Fig 8:**
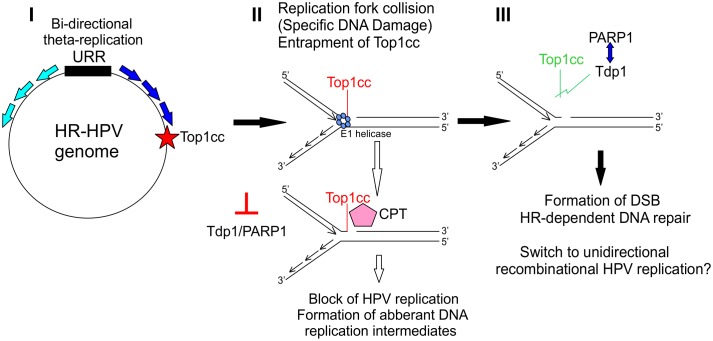
Proposed model for the role of the Tdp1 and PARP1 proteins in high-risk (HR) HPV genome replication. **I:** E1 and E2 dependent bi-directional replication is initiated from the HPV replication origin in the URR region. **II:** Topoisomerase I cleaving complex (Top1cc) will be entrapped in at least one site in the HR-HPV genome due to the specific DNA damage or replication fork collapse. **III:** Tdp1, which is activated through PARylation by the PARP1 protein, cleaves the phosphodiester bond between Top1cc and DNA, thereby releasing the complex. Replication fork collision will be repaired by homologous recombination-dependent DNA repair (using a homologous region from another HPV genome molecule), and HPV genome replication will proceed via unidirectional recombination-dependent replication.

It has become clear in recent years that HPVs activate the DNA damage response network during their replication to “invite” cellular replication and DNA repair proteins to their replication foci[50]. Moreover, it has been shown that HPV uses homologous recombination to efficiently replicate its genome[39,40]. The exact mechanism and all the necessary cellular partners are not yet known. Inhibitors targeting cellular DDR proteins are valid candidates for cancer therapy; for example, the PARP1 inhibitor ABT-888 is in clinical trials[70]. Two of the compounds (NSC 88915 and NSC 305831, also known as Furamidine) identified in this study are known inhibitors of the DDR network protein Tdp1[55,56]. Tdp1 is not an absolutely essential protein for normal cellular replication; however, it is needed for repairing certain types of DNA damage. Due to specific DNA damage or replication fork collapse, Topoisomerase 1 and 2 cleaving complexes (Top1/2cc) become entrapped on the DNA and will interfere with normal replication/transcription fork progression. Tdp1 releases these entrapped Top1cc and Top2cc proteins from the DNA[71,72]. Inhibitors of Tdp1, together with its activator, PARP1, and topoisomerase I inhibitors are considered viable drugs in cancer therapy[73]. Here, we have shown that Tdp1, together with its regulator/activator protein PARP1, are essential cellular proteins in HPV18 replication, since downregulation of these proteins decreases the HPV genome copy number (Fig 5), thus making those proteins good targets for developing HPV inhibitors. Moreover, as Tdp1 and PARP1 are necessary for cancer cell survival, their inhibitors may also be effective against HPV-induced cancer. Thus, the same compounds could be used to cure all HPV-related cancers. Camptothecin (CPT) is a Topoisomerase I inhibitor that stabilizes the interaction between Topoisomerase and DNA[59,60]. Thus, by simultaneously inhibiting Tdp1/PARP1 and using CPT to further stabilize the entrapped Top1cc, more effective inhibition should be achieved[74]. We show here that four out of five compounds indeed have modest synergistic effects; up to five times more efficient inhibition of HPV18 initial amplification was observed when CPT was supplemented, suggesting that these compounds may inhibit Tdp1 or have some other target related to releasing Top1cc complexes from DNA. The inhibition seems to be relatively specific, as the compounds did not decrease the levels of the EBV EBNA1-dependent replication of the oriP plasmid significantly, which, in contrast to HPV amplification, takes place only once per cell cycle, reminiscent of cellular DNA replication (Fig 6). These results suggest that at some point during HPV replication, entrapment of Top1cc occurs on the viral genome. Tdp1 seems to then be activated (probably by PARP1[57]) and recruited to HPV DNA, where it releases those complexes, allowing replication/transcription to continue. When Tdp1 is inhibited, abnormal replication intermediates could emerge, and HPV replication cannot be completed (a proposed model of the pathway is in Fig 8). A study by Edwards et al. has shown (through siRNA library screening) that instead of Tdp1, Tdp2 might be necessary for HPV replication [25]. We analyzed the effects of Tdp2 downregulation on cell cycle progression of U2OS cells by two different shRNA-s and saw significant enrichment of cells in G1 or G2/M phase of the cell cycle (S8 Fig and S2 Table). Thus, it is difficult to assess the role of Tdp2 in HPV replication specifically. The reduction in HPV copy number by downregulation of Tdp2 shown by Edwards et al. may very well be due to the cell cycle block. Additionally, it is known that both Tdp1 and Tdp2 can release the Top1cc complexes from DNA [66]. It is also possible that in different experiment setups or in different cellular systems, either Tdp1 or Tdp2 are used during HPV replication. Regardless, it is still fascinating how HPV replication machinery uses cellular pathways to facilitate its genome replication.

It is unclear how Tdp1 is recruited for the HPV replication process and if it is necessary for all HPV types. More than 99% of all cervical cancer cases are caused by five HR-HPV types: 18, 16, 31, 33 and 45. We show here that all five inhibitors successfully block the initial amplification of all of those HR-HPV types (Figs 4 and 7). Unexpectedly, however, none of the compounds were able to block the replication of low-risk HPV type 11 or cutaneous beta HPV type 5 (Fig 7A and 7B). It is possible that there is a (minor) difference between the replication mechanism of these viruses. Thus far, the two known major differences between HR and LR HPVs are the sequence of the genome and oncogenic potential of the E6 and E7 proteins, which interact with various DNA repair proteins[75,76]. The latter seems not to be the reason for this difference, as the compound NSC305831 still inhibited HPV18 replication when the oncoproteins E5, E6 and E7 alone or in combination were mutated (S9 Fig). It was also clear that the DDR was activated during the replication of all HPVs tested. Since Top1/2cc-s only become trapped on DNA due to specific DNA damage, the nature of DDR activation could differ between high-risk and other HPV types. In addition to the genome sequence and oncoproteins, the viral replication proteins E1 and E2 may differ between HPV types. For example, similar mutations in the C-terminus of the E1 protein completely abolished BPV1 replication but not HPV11 replication[77]. As none of the inhibitors blocked the E1/E2-dependent replication of the 1-kbp-long HPV origin-containing plasmid URR (S5 Fig), it seems that Tdp1 is not crucial for the initiation of HPV replication. In this “URR-assay”, the levels of E1 and E2 protein are significantly higher compared to the HPV viral genome replication and thus the rate of replication initiation is also higher. This may mean that even if the Top1cc is entrapped on URR plasmid, the high rate of replication may not affect the URR plasmid copy number. However, this does not rule out the possibility that E1 and/or E2 interaction with key proteins in the pathway responsible for the release of Top1cc is critical in the case of 8-kbp HPV genome replication.

In addition to the involvement in replication, the inhibitors described in this study may (also) affect HPV gene expression. It is known that Top1 is necessary to relax tensions in DNA during both replication and transcription. Entrapment of Top1cc affects transcription in several ways, perhaps most important of which in the context of HPV life cycle could be the negative impact on splicing[78–81]. Since there are significant differences in mRNA splicing between LR and HR HPVs (reviewed extensively in [82]), for example LR-HPVs do not express E6*. Thus, entrapment of Top1cc may be more deleterious to oncogenic HPV E1/E2 gene expression. The potential effects on HPV gene expression may be another explanation, why these compounds did not work in the “URR assay” as in this case E1 and E2 proteins are expressed from heterologous expression vectors containing CMV promoter.

Regardless, further analyses should be performed to describe the involvement of Tdp1 and PARP1 in HR-HPV replication or gene expression. It would be interesting to identify the specific location(s) in the HPV genome where Top1cc entrapment occurs. Studying the differences in the involvement of Tdp1 between high-risk, low-risk and cutaneous HPV replication would perhaps describe some yet unknown differences between the replication mechanisms of these viruses. It is possible that aside from E6 and E7, differences in replication could be the reason why HR-HPVs, but not LR-HPVs, integrate more readily into the host genome and are therefore causative agents of various cancers.

Collectively, we have engineered modified HPV genomes that express Renilla luciferase as a marker that could be used to monitor viral replication in various assays. The gene expression and replication properties of these marker genomes are almost identical to *wt* genomes and such genomes could be thus used in primary keratinocytes or other suitable cell lines for rapid HPV genome copy number quantification. We used this system in high-throughput screening and have identified several novel HR-HPV-specific inhibitors. Importantly, we have demonstrated that three compounds (88915, 109128 and 305831) inhibit the HPV replication in the cells derived from human cervical intraepithelial neoplasia (CIN612E; Fig 7). This suggests that the target(s) of these compounds are also active during HPV replication in vivo. Analyses of the inhibitory properties of these compounds led to the discovery of Tdp1 and PARP1 as promising targets for the development of new anti-HPV drugs.

When Tdp1 and/or PARP1 are inhibited and Top1cc is stabilized by Camptothecin (CPT), replication forks will collide, HPV genome replication is blocked, and eventually, aberrant DNA replication intermediates form.

## Supporting information

S1 Fig Description of the modified U2OS GFP2-Fluc #10.15 cell-line. **A:** U2OS GFP2-Fluc #10.15 cell-line is suitable for studying the replication of HPVs. U2OS wt or #10.15 cells were transfected with 2 μg of wt HPV18 minicircle genome. The genomic DNA was extracted 3 and 5 days after the transfection and digested with BglI to linearize the HPV18 genome and with DpnI to eliminate bacterially produced input DNA. Replication signals were quantitated by qPCR and are expressed relative to the U2OS 3-day timepoint, as described in the Materials and Methods section. **B:** Firefly luciferase expression by U2OS GFP2-Fluc #10.15 cells resembles cell growth. A total of 150 000 U2OS wt or #10.15 cells were seeded onto 6-well plates and grown for 3 and 5 days. The Fluc levels were measured and are expressed relative to the 3-day timepoint of #10.15 cells. U2OS wt served as a negative control. **C:** Flow cytometric analyses of U2OS wt and #10.15 cells, showing homogenous and clearly detectable GFP signal for U2OS #10.15. U2OS wt served as a negative control. Error bars indicate the standard deviations from two independent experiments. Cell growth and viability could be evaluated by the Firefly luciferase and GFP reporter gene expression.
(TIF)

S2 Fig Description of first- and second-generation marker genomes. It was previously shown that the HPV18 genome that lacks a late region (HPV18 “early” genome) replicates similarly to the *wt* genome in U2OS cells. We therefore generated two different generations of HPV marker genomes that contain reporter genes in the late region. **A:** Schematic of a first-generation marker genome. Non-HPV regions are marked in black. **B:** Schematics of the second-generation marker genomes. Non-HPV regions are marked in black. **C:** U2OS cells were transfected with 1 μg of indicated HPV minicircles, the low-molecular-weight DNA was extracted 48, 72 and 96 hours after the transfection, linearized, and bacterially produced input DNA was digested with DpnI. Southern blot analyses were carried out to measure the replication properties of HPV18 *wt* (lanes 1–3), the first-generation marker genome (lanes 4–6) and two versions of the second-generation marker genomes (lanes 7–12) Linear replication and DpnI-digested HPV18 DNA is shown. Since both the first- and second-generation marker genome replication levels are very low, the image on panel C is greatly overexposed. Insertion of the reporter gene cassettes into the late region of the HPV18 genome greatly interferes with the gene expression and/or replication properties, suggesting that altering the late region would be very difficult if even possible.
(TIF)

S3 Fig The expression of Renilla luciferase from the HPV18-Rluc-E2 genome correlates with changes in the viral copy number. To check if the Renilla luciferase levels correlate with the HPV genome copy number, U2OS #10.15 cells were co-transfected with 2 μg of HPV18-Rluc-E2 marker genome minicircle and 500 pmol of different siRNAs as shown. **A:** The genomic DNA was extracted 2 and 3 days after the transfection, HPV DNA was linearized with BglI, and bacterially produced input DNA was digested with DpnI. Replication signals were quantitated by qPCR. The relative numbers were obtained by normalizing the data points to data from the same timepoint from the HPV18-RlucE2 marker genome and transfection with BPV E1 siRNA. **B:** Both Renilla (from HPV marker genome) and Firefly (from U2OS genome) luciferase were measured in a dual-luciferase assay and are expressed as the Rluc/Fluc ratio. The relative numbers are obtained by normalizing the data points to data from the same time point HPV18-RlucE2 marker genome and transfection with BPV E1 siRNA. In both panels, the average values with standard deviations from three independent experiments are shown. **C:** Scheme of HPV18 early region, where positions of the siRNAs are indicated. 83–105 is against the early promoter (p102), 965–987 is against E1 and 3893–3915 is designed against early mRNAs polyadenylated after this sequence. The decrease in the viral copy number is very similar to the reduction of Renilla luciferase expression, and thus it adequately reflects the HPV copy number.
(TIF)

S4 Fig Comparative transcription map analysis of HPV18-RlucE2 and wt HPV18 in U2OS cells. PolyA+ RNA templates were extracted from U2OS cells that had been transfected with 500 ng of the wt HPV18 genome or with HPV18-RlucE2 (72 h time-point). 500 ng of polyA+ RNA were used as a template for 5'RACE with the HPV18-specific primers Pr1397 (binds to E1 ORF) and Pr904-1 (binds to E7 ORF). The promoter regions from which the detected transcripts arisen, are indicated by arrows on the right.
(TIF)

S5 Fig The identified compounds (structures in S7 Fig) do not inhibit HPV18 URR plasmid replication dependent of expression of the E1 and E2 proteins from heterologous expression vectors. U2OS cells were transfected with 25 ng of the expression vectors for the HPV18 E1 and E2 proteins together with 500 ng of the HPV18 URR (origin) minicircle plasmid. The cells were grown in the presence of compounds at the indicated concentrations for 24 or 48 hours, with DMSO serving as a vehicle control. Genomic DNA was extracted at the indicated timepoints, HPV18 URR DNA was linearized with BglI, and bacterially produced input DNA was digested with DpnI. HPV URR replication signals were detected by Southern blot analyses. Compound 88915 seems to have a positive effect on HPV18 URR replication. It is possible that this compound has a positive effect on cell growth (prolonged S-phase, for example), or it may stabilize (HPV) replication proteins.
(TIF)

S6 Fig Effect of compounds on the stable maintenance and late amplification of the HPV18 genome. The monoclonal U2OS cell line #1.13 contains an episomal HPV18 genome, and its replication and gene expression properties are similar to other HPV-positive cell lines. When maintained in sub-confluent conditions, the stable maintenance phase of HPV18 can be monitored. Confluent conditions, however, seem to mimic the differentiation process of keratinocytes, and thus late amplification of the HPV genomes occur. **A:** U2OS #1.13 cells were grown in sub-confluent conditions in the presence of compounds for 7 days. Genomic DNA was extracted, HPV DNA was linearized with BglI, and the viral replication signal was detected by Southern blot and **B:** quantified by phosphoimager. **C:** U2OS #1.13 cells were seeded and grown for 5 days without splitting. A clear rise in the replication level occurred between the 0-day and 5-day timepoints (compare lanes 17 and 18), showing that the switch to late amplification replication occurred. On the 5^th^ day, when late amplification had been turned on, the indicated concentrations of the compounds were added to the media, and the cells were grown for an additional 7 days without splitting. On the 12^th^ day, genomic DNA was extracted, HPV DNA was linearized with BglI, and the viral replication signal was detected by Southern blot and **D:** quantified by phosphoimager. Error bars represent standard deviations from three independent experiments. Compounds 82269, 88915, 109128 and 305831 (lanes 4–14 in panel **A)** clearly inhibit the stable maintenance phase of HPV18 replication. Compounds 82269, 109128 and 305831 (lanes 4–6; 10–15 on panel **C)** completely abolish the late amplification of HPV18 genome. Compound 9782 does not inhibit these later stages of HPV18 replication. All the quantified signals are expressed relative to the vehicle control, DMSO, and error bars represent standard deviations from two different experiments. The compound 88915 and 305831 should have identical target (Tdp1) but the 88915 does not inhibit the late amplification of HPV18 in U2OS cells. It may result from the fact that this protein has very low potency and more effective inhibition of Tdp1 is required to inhibit the rapidly ongoing late amplification. The compound 9782 should also inhibit the stable maintenance and late amplification as it acts like a Tdp1 inhibitor by showing synergistic inhibition with CPT (Fig 6). However, this compound may actually not inhibit Tdp1 itself but some related protein in this pathway which is dispensable during the later stages of HPV replication.
(TIF)

S7 Fig Summary of the compounds identified in this study. Given are name of the compound, its 2D structure, its activities and its expected target molecule.
(TIF)

S8 Fig Representative cell cycle profiles showing that the compounds identified in this study do not block the cell cycle progression. HPV18-transfected (**A**) or non-transfected U2OS cells (**B**) were plated onto 6-well plates and grown in the presence of the compounds for five days. Cell cycle analyses was performed as described in the materials and method section. Results are also shown in S1 Table. **C**: U2OS cells were transfected with shRNAs specific against Tdp1, Tdp2 or PARP1, grown for three days and cell cycle profile was analyzed. Compounds nor downregulation of Tdp1 and PARP1 showed no significant changes in the cell cycle progression. By contrast, downregulation of Tdp2 with two different shRNAs caused the cell cycle block in G2/M or G1 phases. Results are also shown in S2 Table.
(TIF)

S9 Fig Compound NSC 305831 inhibits the initial amplification of HPV18 genome that lacks the expression of oncoproteins E5, E6 and E7. It is known that high and low risk HPVs differ from each other due to the different oncogenic potential of E6 and E7 proteins. When we saw that all the compounds identified in this study were specific high risk HPV inhibitors (Fig 7), we first thought that the specificity comes from differences in oncoproteins. To analyze this, HPV18 mutant genomes lacking the expression of E5, E6, E7 or both E6 and E7 were used. The hypothesis was that if the HPV oncoproteins are the reason why the compounds identified were high risk HPV specific, these would not inhibit the replication of oncoprotein mutant genomes. U2OS cells were transfected with indicated HPV18 wt, E5-, E6-, E7- or E6-E7- genomes and indicated concentrations of compound NSC305831 were added to the media, DMSO (D) was used as vehicle control. The cells were grown for 5 days and replication assay performed. The compound NSC305831 inhibited the replication of all different HPV18 genomes used similarly. This result demonstrates that the reason these compounds (or at least compound NSC 305831) do not inhibit the replication of low risk or beta HPVs is not due to the differences in HPV oncoproteins E5, E6 or E7.
(TIF)

S1 Table Table showing the effects of the compounds identified in this study on cell cycle progression of HPV18-transfected and non-transfected U2OS cells. (XLSX)

S2 Table Table showing the effects of Tdp1, PARP1 and Tdp2 downregulation on cell cycle progression. (XLSX)
