# Supplementary figures and images for: Identification of several high-risk HPV inhibitors and drug targets with a novel high-throughput screening assay
Source: PLoS Pathog. 2017 Feb 9;13(2):e1006168. doi: 10.1371/journal.ppat.1006168 (PMC5300127; doi:10.1371/journal.ppat.1006168)

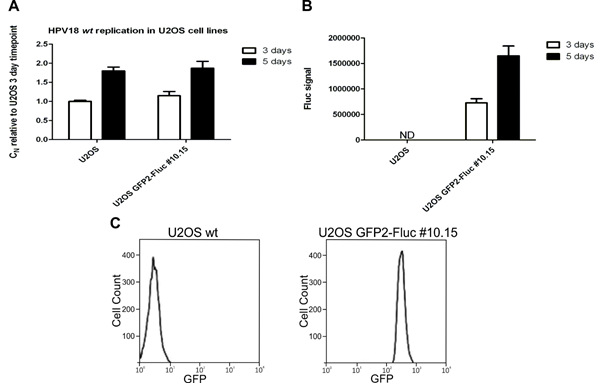

Supplement: S1 Fig — A: U2OS GFP2-Fluc #10.15 cell-line is suitable for studying the replication of HPVs. U2OS wt or #10.15 cells were transfected with 2 μg of wt HPV18 minicircle genome. The genomic DNA was extracted 3 and 5 days after the transfection and digested with BglI to linearize the HPV18 genome and with DpnI to eliminate bacterially produced input DNA. Replication signals were quantitated by qPCR and are expressed relative to the U2OS 3-day timepoint, as described in the Materials and Methods section. B: Firefly luciferase expression by U2OS GFP2-Fluc #10.15 cells resembles cell growth. A total of 150 000 U2OS wt or #10.15 cells were seeded onto 6-well plates and grown for 3 and 5 days. The Fluc levels were measured and are expressed relative to the 3-day timepoint of #10.15 cells. U2OS wt served as a negative control. C: Flow cytometric analyses of U2OS wt and #10.15 cells, showing homogenous and clearly detectable GFP signal for U2OS #10.15. U2OS wt served as a negative control. Error bars indicate the standard deviations from two independent experiments. Cell growth and viability could be evaluated by the Firefly luciferase and GFP reporter gene expression. (TIF) [file ppat.1006168.s001.tif]

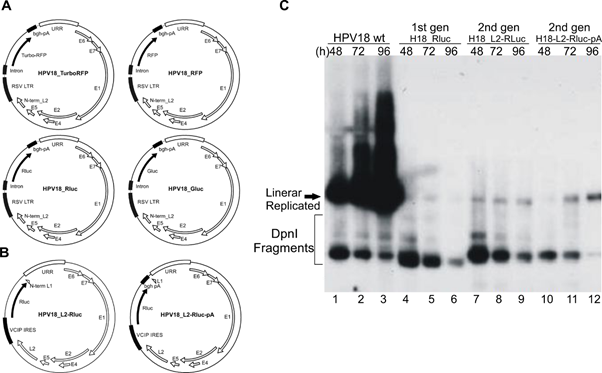

Supplement: S2 Fig — It was previously shown that the HPV18 genome that lacks a late region (HPV18 “early” genome) replicates similarly to the wt genome in U2OS cells. We therefore generated two different generations of HPV marker genomes that contain reporter genes in the late region. A: Schematic of a first-generation marker genome. Non-HPV regions are marked in black. B: Schematics of the second-generation marker genomes. Non-HPV regions are marked in black. C: U2OS cells were transfected with 1 μg of indicated HPV minicircles, the low-molecular-weight DNA was extracted 48, 72 and 96 hours after the transfection, linearized, and bacterially produced input DNA was digested with DpnI. Southern blot analyses were carried out to measure the replication properties of HPV18 wt (lanes 1–3), the first-generation marker genome (lanes 4–6) and two versions of the second-generation marker genomes (lanes 7–12) Linear replication and DpnI-digested HPV18 DNA is shown. Since both the first- and second-generation marker genome replication levels are very low, the image on panel C is greatly overexposed. Insertion of the reporter gene cassettes into the late region of the HPV18 genome greatly interferes with the gene expression and/or replication properties, suggesting that altering the late region would be very difficult if even possible. (TIF) [file ppat.1006168.s002.tif]

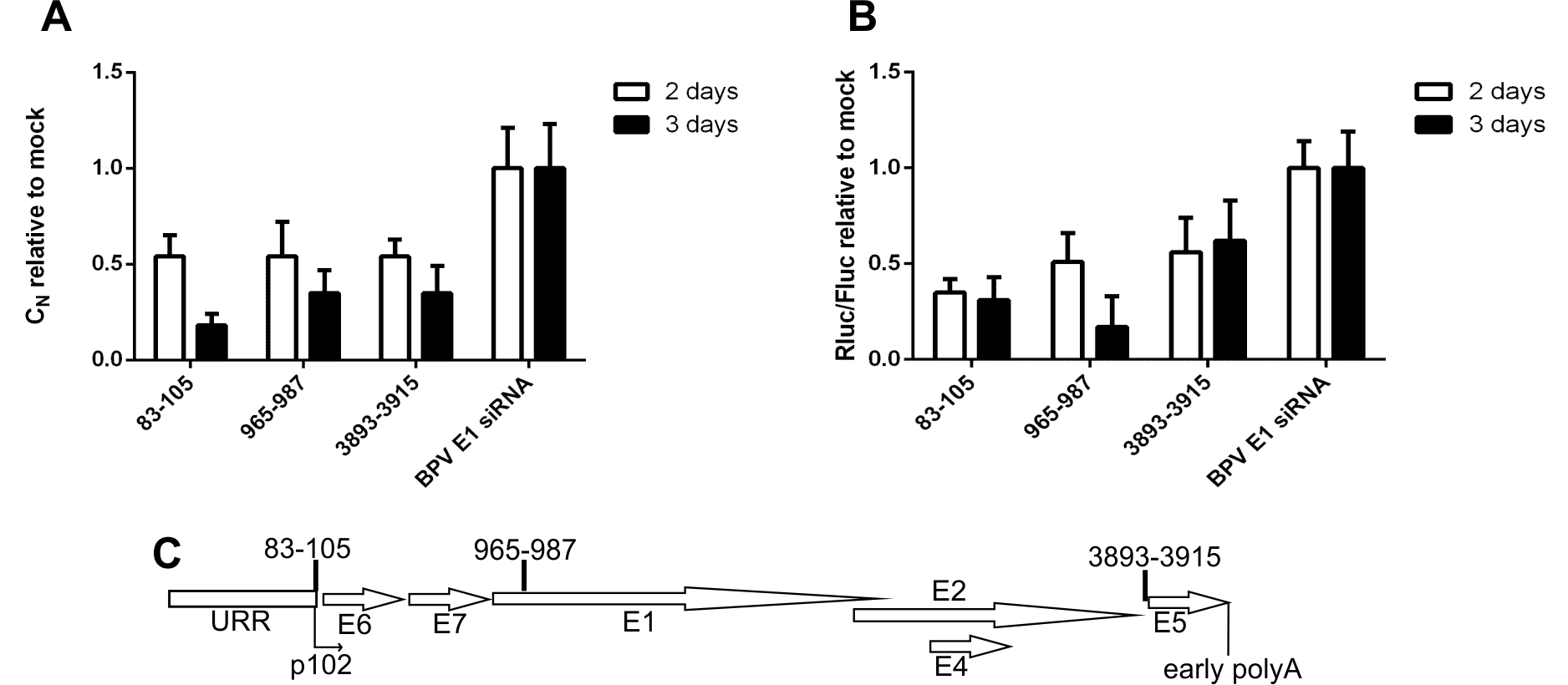

Supplement: S3 Fig — To check if the Renilla luciferase levels correlate with the HPV genome copy number, U2OS #10.15 cells were co-transfected with 2 μg of HPV18-Rluc-E2 marker genome minicircle and 500 pmol of different siRNAs as shown. A: The genomic DNA was extracted 2 and 3 days after the transfection, HPV DNA was linearized with BglI, and bacterially produced input DNA was digested with DpnI. Replication signals were quantitated by qPCR. The relative numbers were obtained by normalizing the data points to data from the same timepoint from the HPV18-RlucE2 marker genome and transfection with BPV E1 siRNA. B: Both Renilla (from HPV marker genome) and Firefly (from U2OS genome) luciferase were measured in a dual-luciferase assay and are expressed as the Rluc/Fluc ratio. The relative numbers are obtained by normalizing the data points to data from the same time point HPV18-RlucE2 marker genome and transfection with BPV E1 siRNA. In both panels, the average values with standard deviations from three independent experiments are shown. C: Scheme of HPV18 early region, where positions of the siRNAs are indicated. 83–105 is against the early promoter (p102), 965–987 is against E1 and 3893–3915 is designed against early mRNAs polyadenylated after this sequence. The decrease in the viral copy number is very similar to the reduction of Renilla luciferase expression, and thus it adequately reflects the HPV copy number. (TIF) [file ppat.1006168.s003.tif]

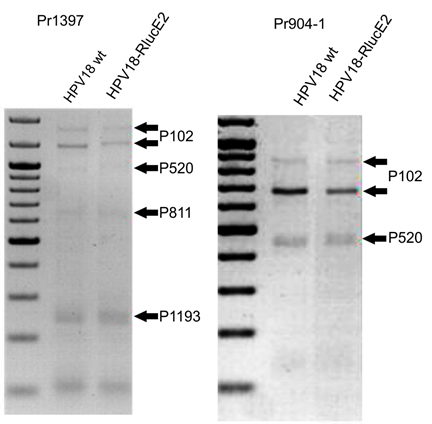

Supplement: S4 Fig — PolyA+ RNA templates were extracted from U2OS cells that had been transfected with 500 ng of the wt HPV18 genome or with HPV18-RlucE2 (72 h time-point). 500 ng of polyA+ RNA were used as a template for 5'RACE with the HPV18-specific primers Pr1397 (binds to E1 ORF) and Pr904-1 (binds to E7 ORF). The promoter regions from which the detected transcripts arisen, are indicated by arrows on the right. (TIF) [file ppat.1006168.s004.tif]

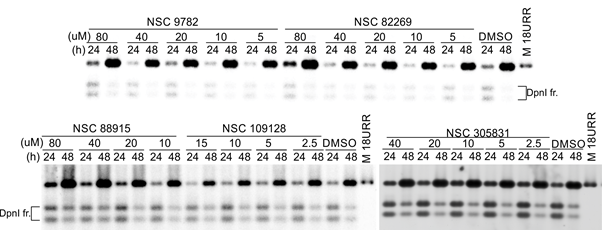

Supplement: S5 Fig — U2OS cells were transfected with 25 ng of the expression vectors for the HPV18 E1 and E2 proteins together with 500 ng of the HPV18 URR (origin) minicircle plasmid. The cells were grown in the presence of compounds at the indicated concentrations for 24 or 48 hours, with DMSO serving as a vehicle control. Genomic DNA was extracted at the indicated timepoints, HPV18 URR DNA was linearized with BglI, and bacterially produced input DNA was digested with DpnI. HPV URR replication signals were detected by Southern blot analyses. Compound 88915 seems to have a positive effect on HPV18 URR replication. It is possible that this compound has a positive effect on cell growth (prolonged S-phase, for example), or it may stabilize (HPV) replication proteins. (TIF) [file ppat.1006168.s005.tif]

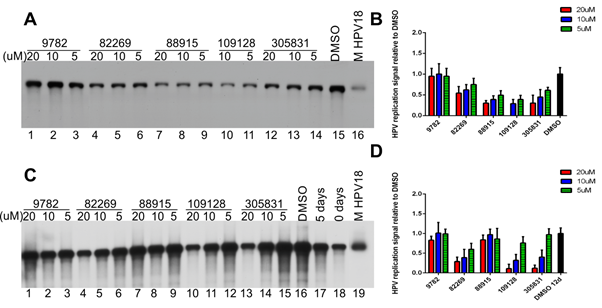

Supplement: S6 Fig — The monoclonal U2OS cell line #1.13 contains an episomal HPV18 genome, and its replication and gene expression properties are similar to other HPV-positive cell lines. When maintained in sub-confluent conditions, the stable maintenance phase of HPV18 can be monitored. Confluent conditions, however, seem to mimic the differentiation process of keratinocytes, and thus late amplification of the HPV genomes occur. A: U2OS #1.13 cells were grown in sub-confluent conditions in the presence of compounds for 7 days. Genomic DNA was extracted, HPV DNA was linearized with BglI, and the viral replication signal was detected by Southern blot and B: quantified by phosphoimager. C: U2OS #1.13 cells were seeded and grown for 5 days without splitting. A clear rise in the replication level occurred between the 0-day and 5-day timepoints (compare lanes 17 and 18), showing that the switch to late amplification replication occurred. On the 5th day, when late amplification had been turned on, the indicated concentrations of the compounds were added to the media, and the cells were grown for an additional 7 days without splitting. On the 12th day, genomic DNA was extracted, HPV DNA was linearized with BglI, and the viral replication signal was detected by Southern blot and D: quantified by phosphoimager. Error bars represent standard deviations from three independent experiments. Compounds 82269, 88915, 109128 and 305831 (lanes 4–14 in panel A) clearly inhibit the stable maintenance phase of HPV18 replication. Compounds 82269, 109128 and 305831 (lanes 4–6; 10–15 on panel C) completely abolish the late amplification of HPV18 genome. Compound 9782 does not inhibit these later stages of HPV18 replication. All the quantified signals are expressed relative to the vehicle control, DMSO, and error bars represent standard deviations from two different experiments. The compound 88915 and 305831 should have identical target (Tdp1) but the 88915 does not inhibit the late amplification of HPV18 in U [file ppat.1006168.s006.tif]

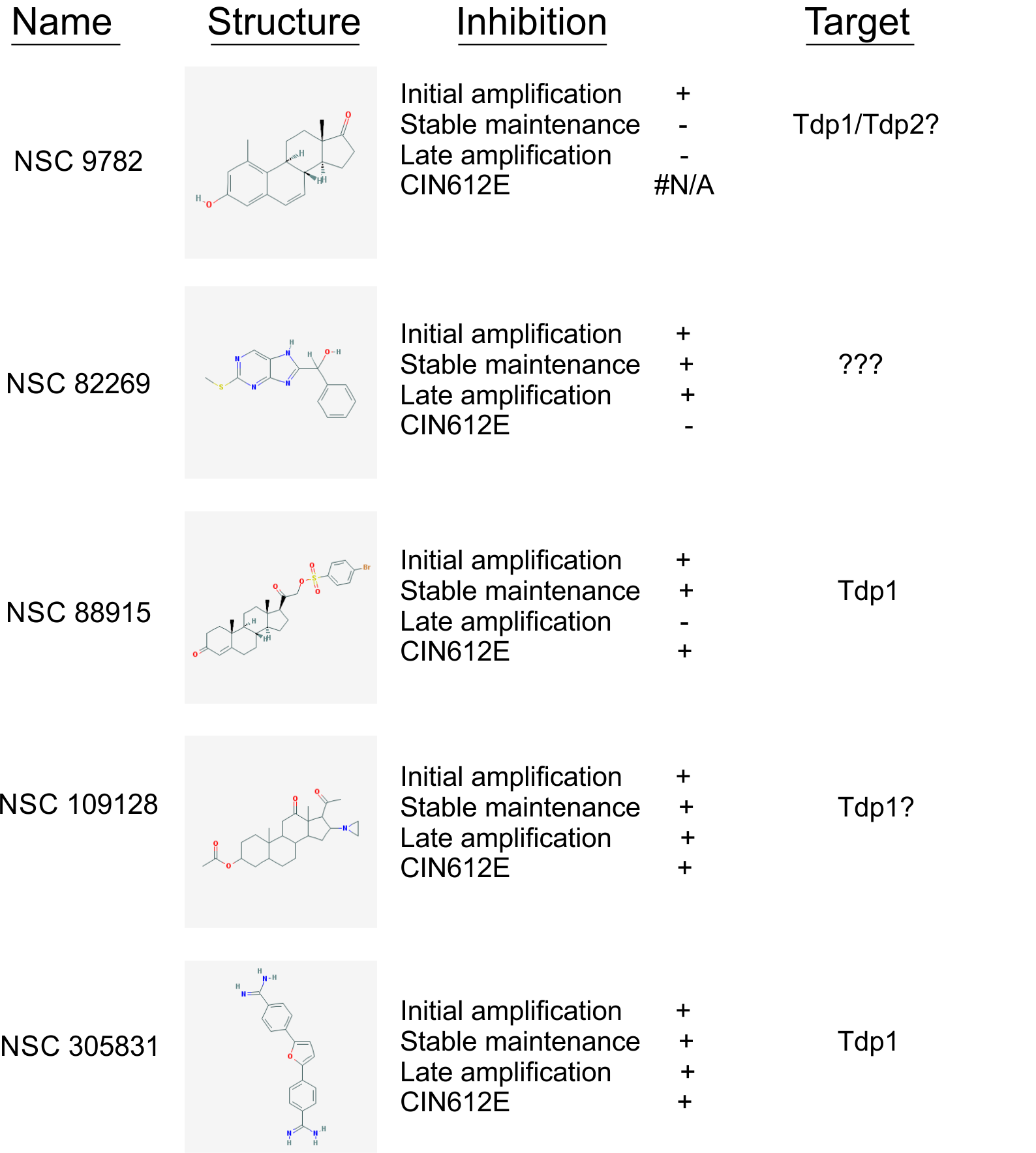

Supplement: S7 Fig — Given are name of the compound, its 2D structure, its activities and its expected target molecule. (TIF) [file ppat.1006168.s007.tif]

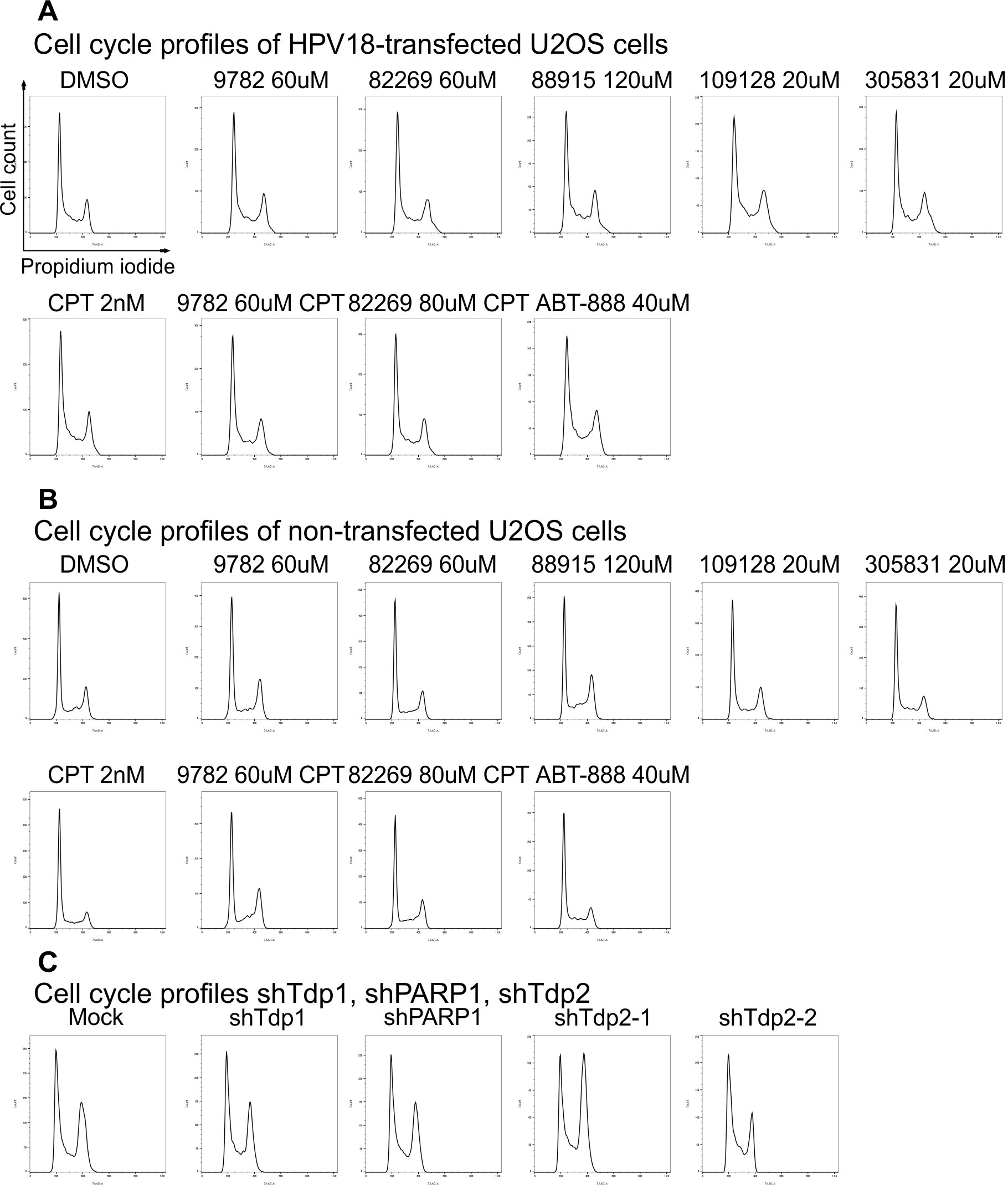

Supplement: S8 Fig — HPV18-transfected (A) or non-transfected U2OS cells (B) were plated onto 6-well plates and grown in the presence of the compounds for five days. Cell cycle analyses was performed as described in the materials and method section. Results are also shown in S1 Table. C: U2OS cells were transfected with shRNAs specific against Tdp1, Tdp2 or PARP1, grown for three days and cell cycle profile was analyzed. Compounds nor downregulation of Tdp1 and PARP1 showed no significant changes in the cell cycle progression. By contrast, downregulation of Tdp2 with two different shRNAs caused the cell cycle block in G2/M or G1 phases. Results are also shown in S2 Table. (TIF) [file ppat.1006168.s008.tif]

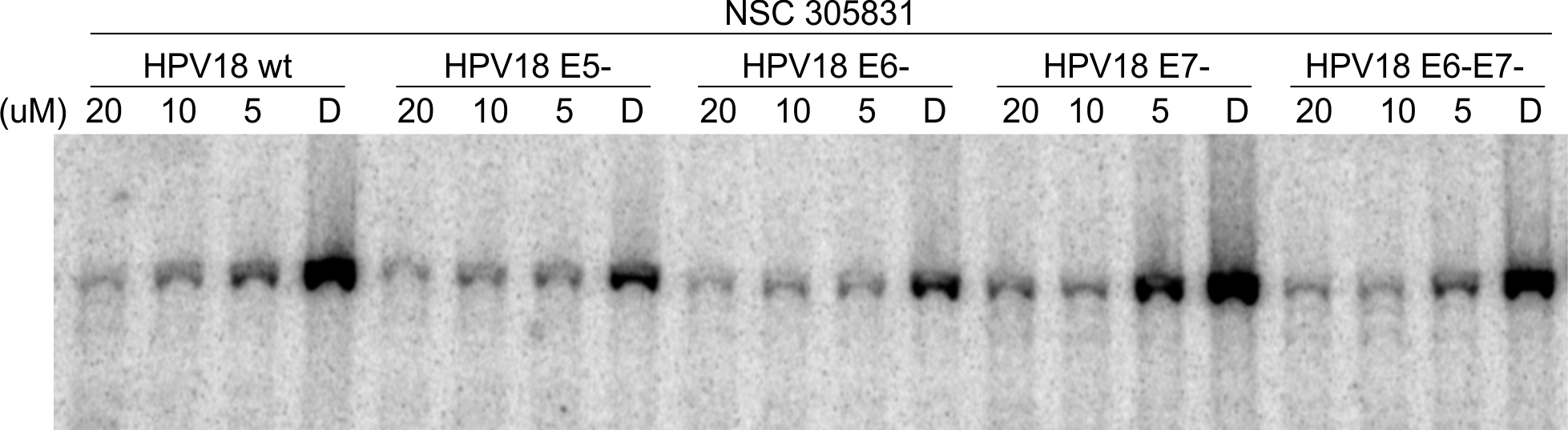

Supplement: S9 Fig — It is known that high and low risk HPVs differ from each other due to the different oncogenic potential of E6 and E7 proteins. When we saw that all the compounds identified in this study were specific high risk HPV inhibitors (Fig 7), we first thought that the specificity comes from differences in oncoproteins. To analyze this, HPV18 mutant genomes lacking the expression of E5, E6, E7 or both E6 and E7 were used. The hypothesis was that if the HPV oncoproteins are the reason why the compounds identified were high risk HPV specific, these would not inhibit the replication of oncoprotein mutant genomes. U2OS cells were transfected with indicated HPV18 wt, E5-, E6-, E7- or E6-E7- genomes and indicated concentrations of compound NSC305831 were added to the media, DMSO (D) was used as vehicle control. The cells were grown for 5 days and replication assay performed. The compound NSC305831 inhibited the replication of all different HPV18 genomes used similarly. This result demonstrates that the reason these compounds (or at least compound NSC 305831) do not inhibit the replication of low risk or beta HPVs is not due to the differences in HPV oncoproteins E5, E6 or E7. (TIF) [file ppat.1006168.s009.tif]
